# Improved vacuum-evaporated blue perovskite light-emitting diodes with phenethylammonium chloride and guanidinium bromide synergistic post-processing modification

**DOI:** 10.1007/s12200-025-00152-8

**Published:** 2025-03-24

**Authors:** Liang Sun, Xiping He, Zhiyuan He, Feihu Zhang, Chencheng Peng, Ben Chen, Runda Guo, Lei Wang

**Affiliations:** Wuhan National Laboratory for Optoelectronics, School of Optical and Electronic Information, Huazhong University of Science and Technology, Wuhan, 430074 China

**Keywords:** Perovskite light-emitting diodes, Blue, Heterojunctions, Vacuum deposition, Post-processing strategy

## Abstract

Metal halide perovskites have become one of the most competitive new-generation optoelectronic materials due to their excellent optoelectronic properties. Vacuum evaporation can produce high-purity and large-area films, leading to the wide application of this method in the semiconductor industry and optoelectronics field. However, the electroluminescent performance of vacuum-evaporated perovskite light-emitting diodes (PeLEDs) still lags behind those counterparts fabricated by solution methods. Herein, based on vacuum evaporation, 3D perovskite films are obtained by three-source co-evaporation. Considering the unique quantum well structure of quasi-2D perovskite can significantly enhance the exciton binding energy and improve the radiative recombination rate, leading to a high photoluminescence quantum yield (PLQY). Subsequently, the highly stable and low-defect-density quasi-2D perovskite is introduced into 3D perovskite films through post-treatment with phenethylammonium chloride (PEACl). To minimize the degradation of film quality caused by PEACl treatment, a layer of guanidinium bromide (GABr) is vacuum evaporated on top of PEACl treatment to further improve the quality of emitting layer. Finally, under the synergistic post-processing modification of PEACl and GABr, blue PeLEDs with a maximum external quantum efficiency (EQE) of 6.09% and a maximum brightness of 1325 cd/m^2^ are successfully obtained. This work deepens the understanding of 2D/3D heterojunctions and provides a new approach to construct PeLEDs with high performance.

**Graphical abstract:**

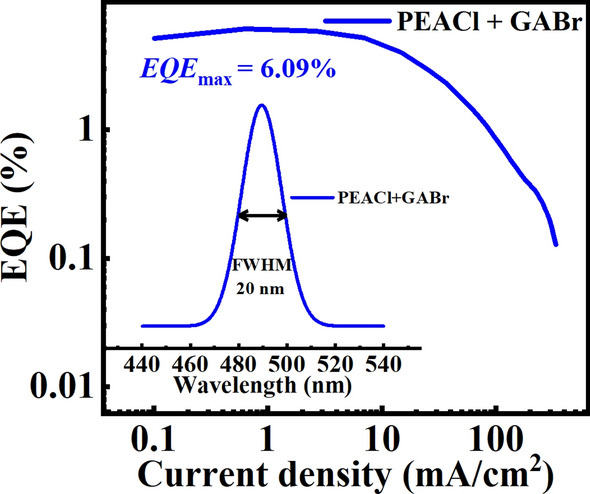

**Supplementary Information:**

The online version contains supplementary material available at 10.1007/s12200-025-00152-8.

## Introduction

PeLEDs based on metal halide perovskite materials exhibit significant prospects for applications in the fields of display technology due to their narrow emission half-peak width, tunable emission colors, high defect tolerance, and low production costs [1–9]. Considering the display application demands for arrayed and pixelated light-emitting devices and the difficulty of heterogeneously integrating display chips with thin film transistor (TFT) or complementary metal oxide semiconductor (CMOS) driving circuit chips [10], vacuum evaporation is a highly valuable route for fabricating PeLEDs [11], which enables precise controlling of the growth rates of precursor materials through crystal oscillator detection systems. Based on these advantages, vacuum-evaporated PeLEDs has become a competitive potential technical route sheds light on the development of high performance display technologies [12].

Although the vacuum evaporation has certain advantages, there is currently limited research on it, and most of the research is focused on green vacuum-evaporated PeLEDs [13]. The study on blue PeLEDs lags obviously behind [14]. During the evaporation process of fabricating blue perovskite emission layers, issues such as defect density and crystal quality often lead to a decline in device performance, resulting in challenges such as low efficiency, low brightness and poor stability [15–17]. These problems have caused significant delays in the development and application of vacuum-evaporated blue PeLEDs [18]. Therefore, developing new strategies to optimize the blue perovskite emission layer plays a significant role in achieving high-performance blue PeLEDs, which is crucial for realizing full-color displays utilizing low cost metal halide perovskite materials [19].

Here in, we adopted a 2D/3D heterojunction structure combined with post-process strategy, successfully enhancing the performance of the blue perovskite layer. After obtaining 3D perovskite films through a three-source co-evaporation process, we introduced a quasi-2D perovskite film on the surface of the 3D perovskite film by PEACl post-treatment to conform a 2D/3D heterojunction. Simultaneously, to reduce the increase in surface roughness caused by the introduction of the 2D/3D heterojunction, a layer of GABr was deposited on the heterojunction surface, which improved the film quality of the perovskite emission layer. As a result, we ultimately obtained a high-performance electroluminescent device with a maximum EQE of 6.09% and a maximum brightness of 1325 cd/m^2^. This research not only advances the development of blue light perovskite materials, but also provides new ideas and directions for the realization of efficient light-emitting devices in the future.

## Results and discussion

### Fabrication of 2D/3D heterojunctions

**Fig. 1:**
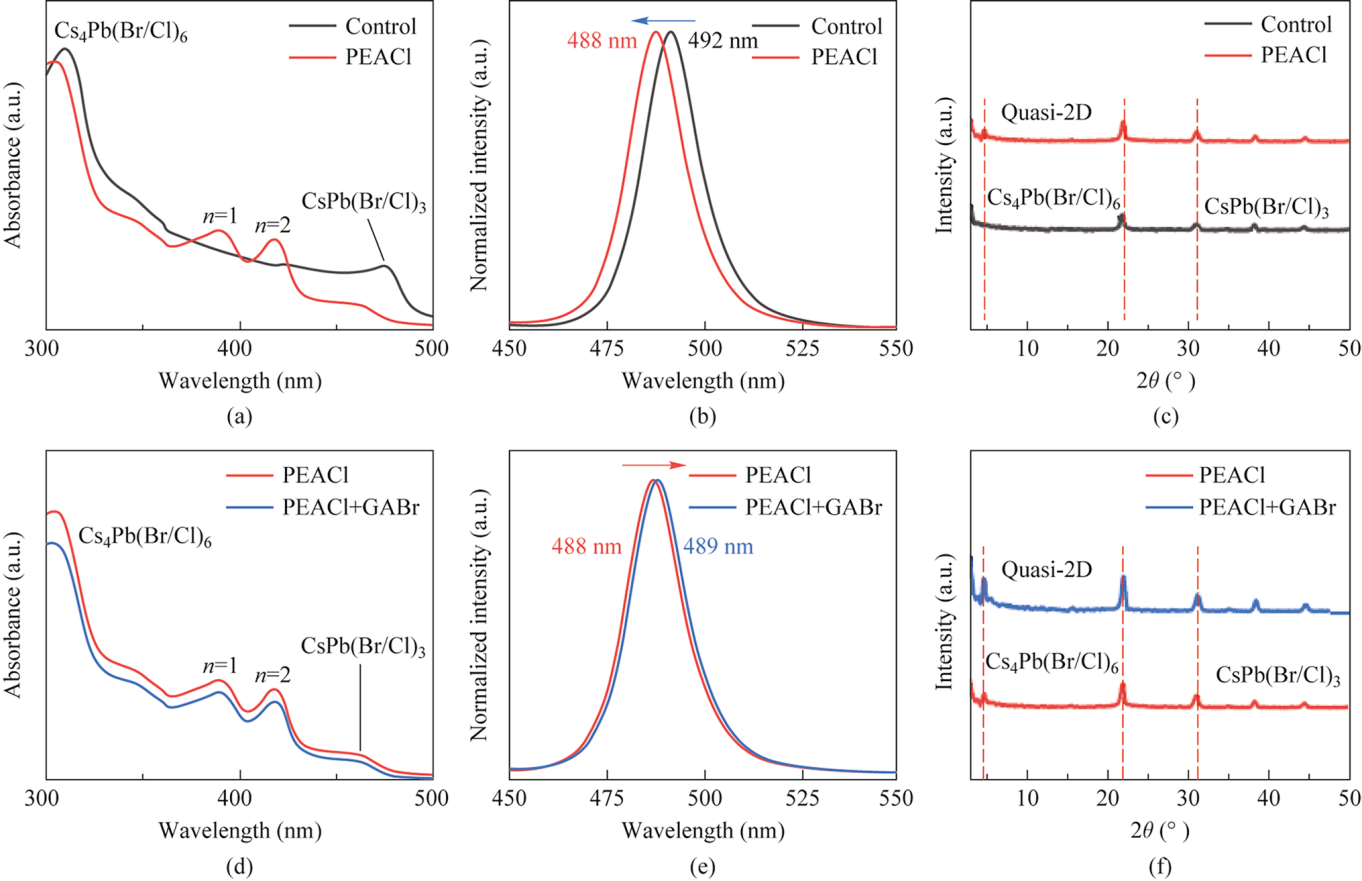
**a** The absorption spectra of perovskite films before and after PEACl treatment. **b** The PL spectra of perovskite films before and after PEACl treatment. **c** The XRD of perovskite films before and after PEACl treatment. **d** The absorption spectra of perovskite films before and after GABr treatment. **e** The PL spectra of perovskite films before and after GABr treatment. **f** The XRD patterns of perovskite films before and after GABr treatment

The perovskite film was prepared by vacuum evaporation, and the preparation process is shown in Fig. S1. Figure 1a shows the absorption spectra of perovskite films before and after PEACl treatment. Perovskite films obtained directly via triple-source co-evaporation (original film) only exhibited two absorption peaks at 480 nm (corresponding to the 3D CsPb(Br/Cl)_3_) and 310 nm (corresponding to the 0D Cs_4_Pb(Br/Cl)_6_) [20]. After PEACl-treatment, two new 2D absorption peaks at 390 nm and 410 nm (corresponding to the *n* = 1 and *n* = 2 in quasi-2D absorption peak, respectively) could be observed, indicating that quasi-2D perovskite phases were successfully obtained through the post-treatment [21]. Meanwhile, the absorption intensity of CsPb(Br/Cl)_3_ and Cs_4_Pb(Br/Cl)_6_ significantly decreased after PEACl-treatment. This indicates that part of CsPb(Br/Cl)_3_ and Cs_4_Pb(Br/Cl)_6_ transfer from 3 and 0D to quasi-2D after PEACl-treatment, which is consistent with the appearance of absorption peaks of quasi-2D. Additionally, a noticeable blueshift of the peak position was observed, indicating that PEACl successfully facilitated halogen exchange during PEACl-treatment, with some of Cl^−^ in PEACl transferring to CsPb(Br/Cl)_3_ emitting phase. To verify this point, photoluminescence (PL) spectra of perovskite films before and after PEACl treatment were tested. The PL spectra (Fig. 1b) showed that the PL peak was blue-shifted from 492 to 488 nm, which was attributed to successful halogen exchange [22, 23].

To further verify the generation of quasi-2D perovskites, X-ray diffraction (XRD) tests were conducted. As shown in Fig. 1c, the diffraction peaks corresponding to the 0D Cs_4_Pb(Br/Cl)_6_ phase at 22° and the 3D CsPb(Br/Cl)_3_ phase at 31° were observed in the original film [24]. After PEACl treatment, a new XRD feature peak appeared below 5° in the XRD spectrum, which is attributed to the quasi-2D perovskite [25]. Figure S2 is the magnification of the XRD patterns for perovskite films. It can be seen that with the increase in Cl content, the diffraction peaks shift to higher angles, further confirming the halide exchange [26]. The results are consistent with the previous absorption spectra.

Since the PEACl-treatment is accompanied by the disappearance of existing crystals and the generation of new crystals, it inevitably leads to significant changes in the morphology of the film. To investigate this change, scanning electron microscopy (SEM) tests were performed. The results (Fig. S3) revealed the surface crystals in the original film exhibited two distributions: small crystals with diameters ranging from 20 to 30 nm and larger crystals with diameters ranging from 100 to 200 nm. It is speculated that the smaller crystals correspond to 3D CsPb(Br/Cl)_3_, while the larger crystals are likely 3D CsPb(Br/Cl)_3_ wrapped by 0D Cs_4_Pb(Br/Cl)_6_ [27, 28]. After PEACl treatment, some of the CsPb(Br/Cl)_3_ and Cs_4_Pb(Br/Cl)_6_ transitioned into quasi-2D, resulting in a noticeable reduction in the size of the surface crystals [29]. Additionally, PEACl treatment led to the formation of voids in the film. These voids are usually considered to be non-radiative recombination centers, which would reduce the PLQY.

### Modification of the perovskite films morphology

To solve the problem of film quality decline which resulting from PEACl treatment, we propose a method of post depositing a GABr buffer layer onto PEACl-treated films. As illustrated in Fig. S3, GABr can fill the voids between the grains effectively, making the film surface denser. This significantly reduces issues such as charge carrier escape and non-radiative recombination at interfaces between perovskite layers and electron transport layers due to inadequate contact [30, 31].

Simultaneously, to further confirm the effects of GABr treatment, atomic force microscopy (AFM) testing was performed. Figure S4 showed that the coexistence of small CsPb(Br/Cl)_3_ crystals and large Cs_4_Pb(Br/Cl)_6_ crystals led to a high root-mean-square (RMS), with a RMS roughness of 4.372 nm. After PEACl treatment, crystal dissolution and new crystal formation slightly increased RMS to 4.962 nm. After GABr treatment, the surface voids were filled and the RMS was reduced to 3.465 nm. This result is consistent with SEM results and demonstrates that GABr treatment can effectively suppress the degradation of film quality caused by PEACl treatment.

To further investigate the impact of GABr treatment on the phase distribution, absorption spectrum tests were conducted. As shown in Fig. 1d, it can be seen that after GABr treatment, there are still absorption peaks located at 310, 390, 420, and 465 nm in the film, indicating that GABr treatment does not significantly affect the phase distribution [32]. To further support this point, the XRD spectra of the film before and after GABr treatment were measured. As depicted in Fig. 1f, the GABr treatment does not affect the characteristic diffraction peaks of each phase in the perovskite film, which is in good agreement with the absorption results.

To study the impact of GABr on photoluminescence, the PL spectra was conducted on the films before and after GABr treatment. As shown in Fig. 1e, the PL peak of the film red-shifted slightly from 488 to 489 nm after GABr treatment, which is the result of halogen exchange and is consistent with the absorption spectrum results. The above results collectively indicate that, unlike PEACl treatment, GABr treatment does not significantly affect the perovskite crystal phase due to the lack of solution assistance during the GABr treatment process.

**Fig. 2:**
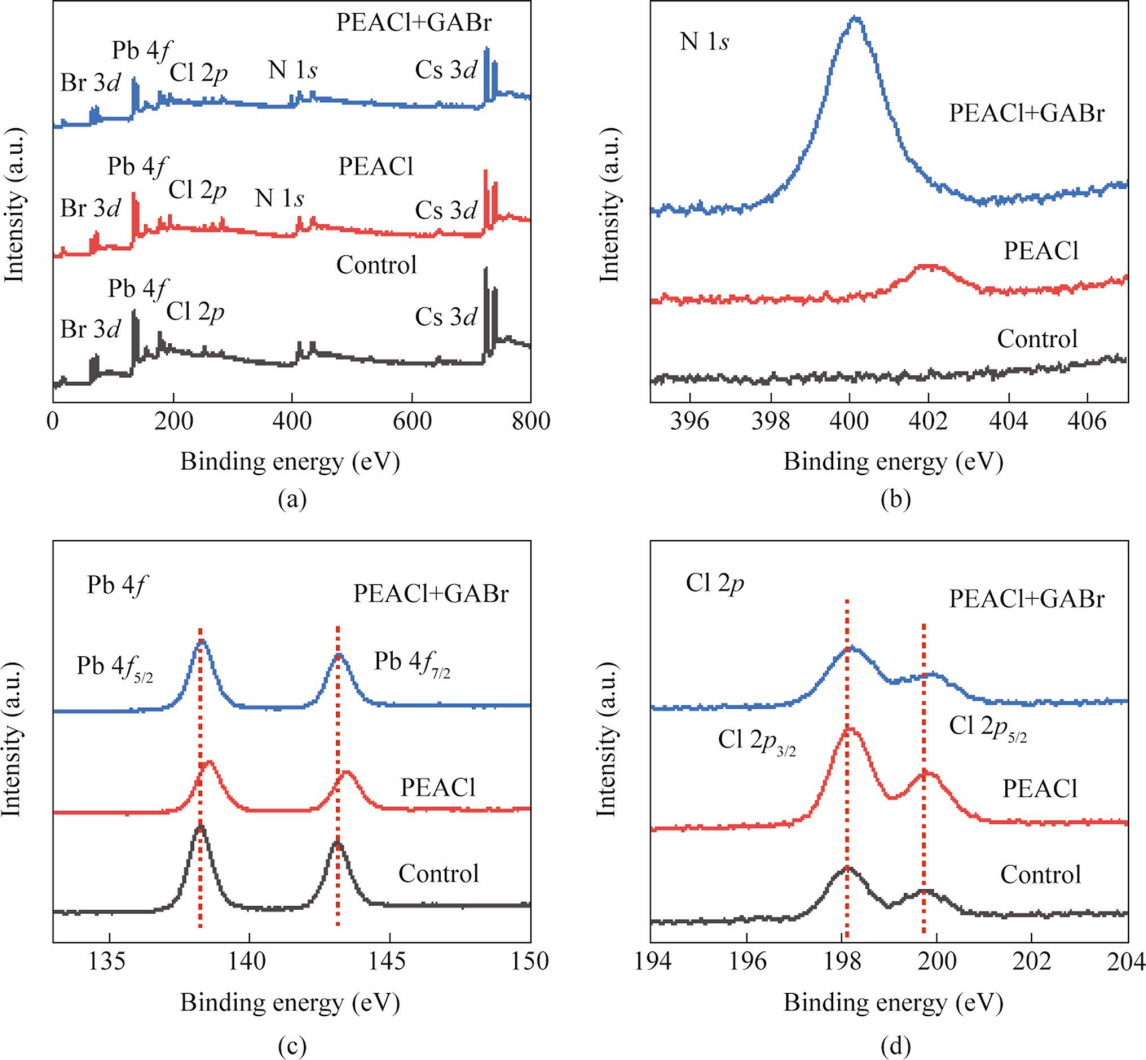
The XPS of perovskite films before and after PEACl and GABr treatments for **a** full spectrum, **b** N 1*s* narrow spectrum, **c** Pb 4*f* narrow spectrum and **d** Cl 2*p* narrow spectrum

To investigate the mechanisms of PEACl and GABr treatments, X-ray photoelectron spectroscopy (XPS) tests were conducted on the original film, the PEACl-treated film and the GABr-treated film. As shown in XPS spectra (Fig. 2a), after PEACl and GABr treatments, characteristic peaks belonging to N 1*s* appeared around 400 eV, indicating that N element was successfully introduced into the film. The narrow-spectrums of the characteristic peaks for each film further reflected the experimental results. The narrow-spectrum of N 1*s* (Fig. 2b) show that N element characteristic peaks begin to appear in the film after PEACl treatment, indicating that PEA^+^ has been successfully introduced into the film after PEACl treatment. After GABr treatment, the peak position of N 1*s* characteristic peak shifted. Additionally, the chemical environment of the N element is different from that of the N element in PEACl, indicating the successful introduction of GA^+^. In general, the prepared perovskite films have many defect [33]. The uncoordinated Pb^2+^ is the most common defect and also the primary non-radiative recombination center. As shown in Fig. 2c, after PEACl treatment, the XPS characteristic peaks of Pb 4*f*_5/2_ and Pb 4*f*_7/2_ moved from 138.27 and 143.07 eV to higher binding energies of 138.59 and 143.39 eV, respectively. This shift is attributed to the formation of Pb-X halide bonds during PEACl treatment, which is consistent with the trend of Cl 2*p*_3/2_ and Cl 2*p*_5/2_ XPS characteristic peaks in the Cl 2*p* from 198.09 and 199.71 eV toward higher binding energies of 198.17 and 199.81 eV (Fig. 2d), respectively [34]. In contrast, after GABr treatment, the XPS characteristic peaks of Pb 4*f*_5/2_ and Pb 4*f*_7/2_ shifted back from 138.59 and 143.39 eV to lower binding energy of 138.27 and 143.07 eV, which indicates that GABr interacts with Pb^2+^ to supply electrons, thereby reducing defect density and suppressing non-radiative recombination [35]. Specifically, The -NH_2_^+^ contained in GA^+^ has a certain electron supply effect, and by providing electrons to the uncoordinated Pb^2+^, the electron cloud of Pb^2+^ can be modified to some extent. Meanwhile, the N–H group interacts with Pb^2+^ to realize the passivation effect [36, 37].

**Fig. 3:**
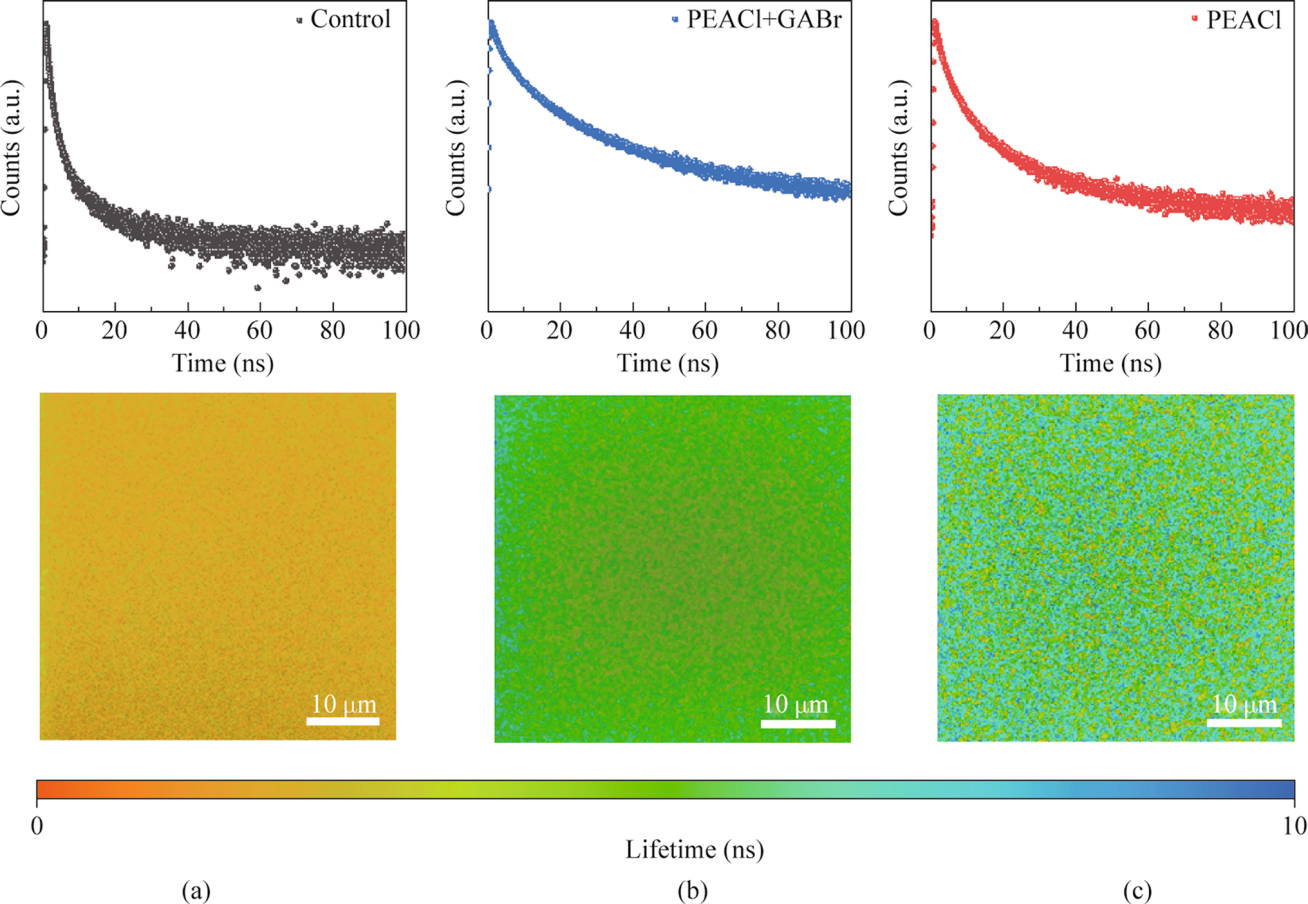
The TRPL spectra and mapping of perovskite films for **a** control, **b** PEACl-modified and **c** PEACl and GABr treatments

Furthermore, the impact of post-treatment on defect density was confirmed by time-resolved photoluminescence (TRPL) tests. Figure 3 illustrates the TRPL decay curves of the three types of films. According to the Note S1, the values of *τ*_1_, *τ*_2_, *A*_1_, *A*_2_, and *τ*_ave_ for different samples can be calculated, as summarized in Table S1. After PEACl treatment, the average carrier lifetime *τ*_ave_ increased from 0.83 to 3.44 ns, indicating a significant reduction in defect density due to the filling of halide vacancies. After GABr treatment, *τ*_ave_ further increased to 7.69 ns, showing an additional decrease in defect density from the passivation of uncoordinated Pb^2+^. TRPL mapping measurements were also performed to reflect the carrier lifetimes across the entire film. The original film showed a shorter lifetime in the orange-red region, which became longer after PEACl treatment (green region), and reached the longest lifetime in the blue-green region after GABr treatment, consistent with the TRPL measurements.

**Fig. 4:**
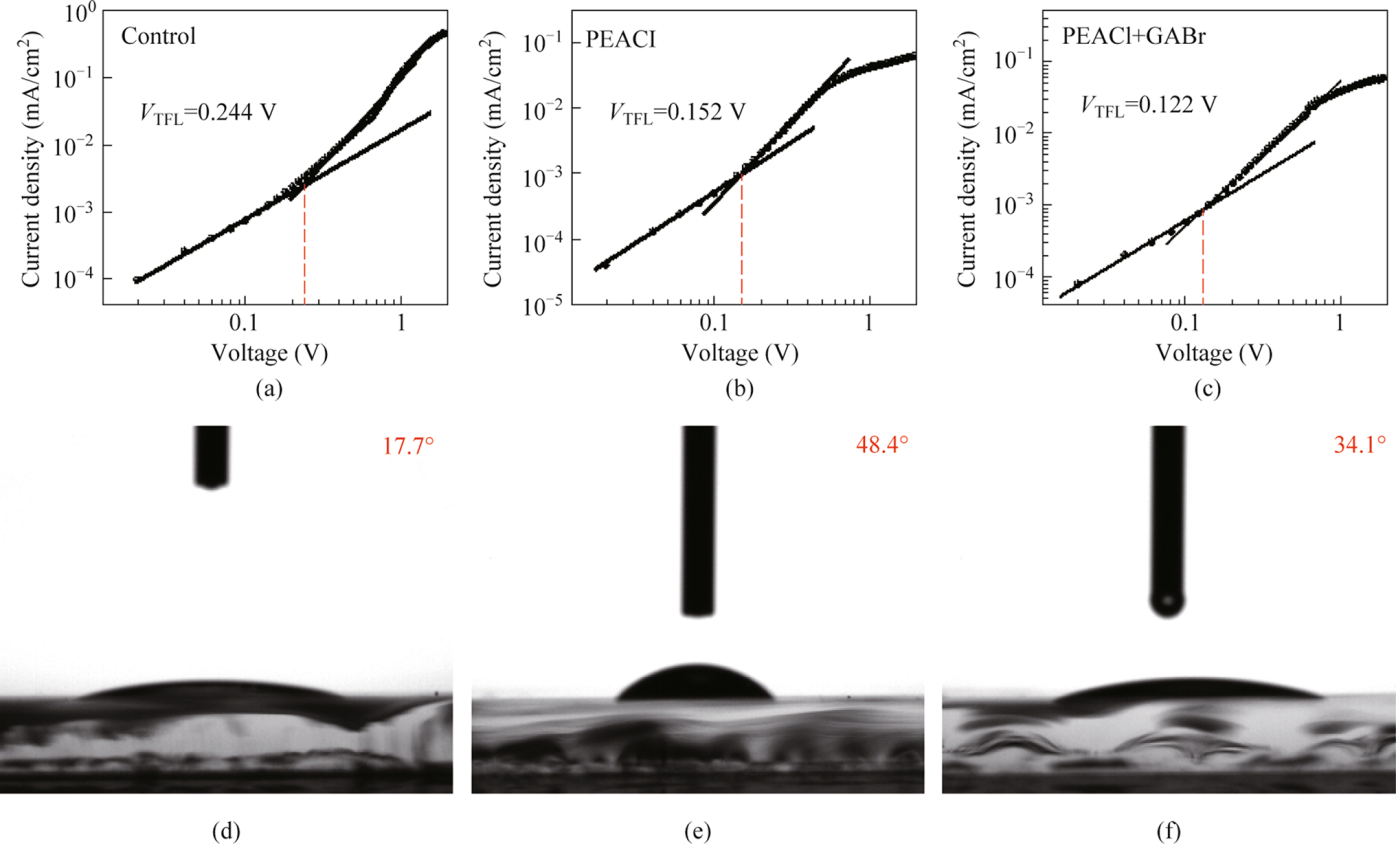
Current density versus voltage characteristic of the PeLEDs for **a** control, **b** PEACl-modified and **c** PEACl and GABr treatments. Water contact angle of films for **d** control, **e** PEACl-modified and **f** PEACl and GABr treatment

To further validate the results of TRPL, space-charge limited current (SCLC) tests were conducted to evaluate their defect densities. Hole-only devices were fabricated with the structure of ITO/NiO_*x*_/polyvinyl carbazole (PVK)/Perovskite/N,N″-Di-[(1-naphthalenyl)-N,N″-diphenyl] − 1,1″-biphenyl) − 4,4″-diamine (NPB)/MoO_3_/Al. Particularly, due to the concurrent blue shift of the PL peak during the post-processing, the control group used a perovskite film with a PL peak at 488 nm directly obtained via the three-source co-evaporation process to mitigate the effect of blue shift. The defect density was quantified by analyzing their current − voltage (*J − V*) characteristics at different voltages. As shown in Fig. 4a − c, the *V*_TFL_ of the single-carrier device decreased from 0.244 V for the original film to 0.152 V after PEACl treatment, and further decreased to 0.122 V after GABr treatment. The defect density can be calculated according to the following formula based on the value of *V*_TFL_ [38]:

1 $$n_{\text{trap}}=V_{\text{TFL}}(2\varepsilon\varepsilon_{0})/(ed^2).$$

Here, *ε* and *ε*_0_ represent the sample dielectric constant [39] and the vacuum dielectric constant, respectively, *d* is the thickness of the perovskite film, and *e* is the charge of an elementary electron. According to Eq. (1), the defect density of the perovskite film after PEACl treatment decreased from the original 6.47 × 10^15^ to 4.03 × 10^15^ cm^−3^, and further decreased to 3.23 × 10^15^ cm^−3^ after GABr treatment. This indicates that both post-treatment processes can effectively reduce the defect density, which is consistent with the TRPL results. Benefiting from the defect passivation effect of the post-treatment, the PLQY of the obtained films increased from the original 13.5% to 24.1% after PEACl treatment and finally reached 39.7% with the effect of GABr.

Generally, perovskite is sensitive to moisture, especially degradation in high humidity environments [40]. The moisture resistance of the film was evaluated contact angle testing, depicted in Fig. 4d − f. Post-treatment with PEACl increased the contact angle of the perovskite film from an initial 17.7° to 48.4°, primarily due to the strong hydrophobicity of quasi-2D perovskite [41]. After GABr treatment, the contact angle of the film slightly decreased but overall remained higher compared to the original film, which indicates that the post-processing process can effectively improve the operational stability and moisture resistance of the film.

### Post-treatment effects on PeLEDs performances

In general, post-treatment processes can significantly affect the band alignment at the interface, thereby influencing the carrier transport at the interface. Therefore, ultraviolet photoelectron spectroscopy (UPS) tests (Fig. S5) were conducted to calculate the bandgap of the perovskite layer film after post-treatment. According to Note S3, the increase in the valence band maximum can reduce the hole injection barrier and enhance the hole injection efficiency.

**Fig. 5:**
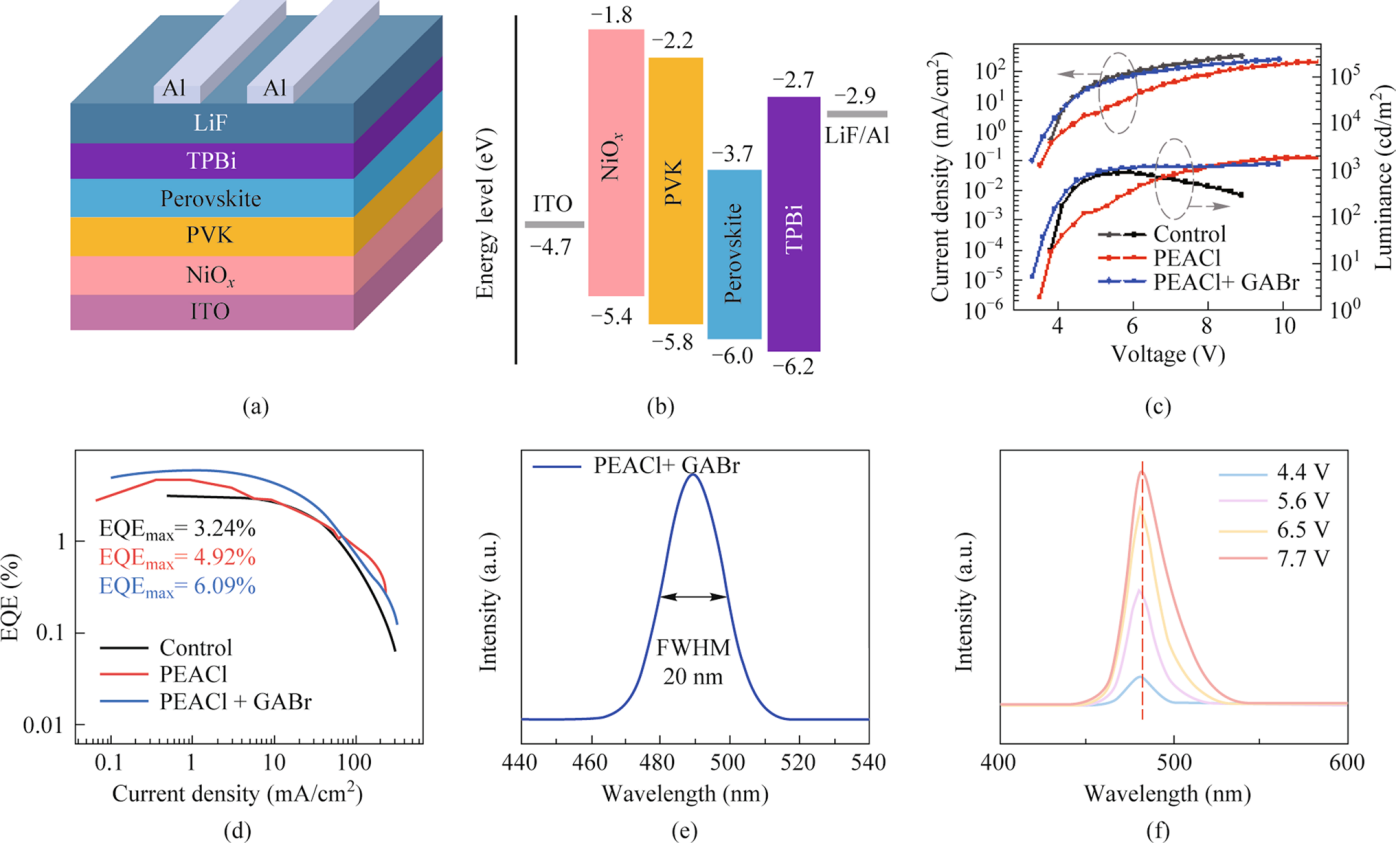
**a** Device architecture of PeLEDs. **b** Energy-level diagram of the device structure. **c** Current density and luminance versus voltage characteristics of PeLEDs. **d** Current density-dependent EQE curves for PeLEDs. **e** EL spectra of post-processed PeLEDs at 4.4 V. **f** EL spectra of post-processed PeLEDs at different voltage bias

Benefiting from the excellent optoelectronic properties of the post-treated film, the PeLEDs with the device structure shown in Fig. 5a were fabricated, consisting of ITO/NiO_*x*_/PVK/Perovskite/TPBi/LiF/Al, and the energy levels for the whole device were shown in Fig. 5b. Figure 5c illustrates the current − voltage − luminance curves (*J − V − L*) for different devices. It is obvious that the maximum luminance (*L*_max_ = 1325 cd/m^2^) of the post-treated PeLEDs is higher than that of the original PeLEDs due to the reduction in defect density and increased hole injection efficiency after post-processing. Among them, PeLEDs treated with PEACl exhibit higher maximum luminance, mainly because GABr is an organic material with a high resistance, which slows down the transmission rate of charge carriers at the interface. Based on Fig. S6, it can be observed that the device treated with PEACl exhibits a relatively larger roll-off, while the device treated with GABr has the smallest roll-off. This is attributed to the reduction in film defect density after GABr treatment. As depicted in Fig. 5d, the maximum EQE of PeLEDs was improved from 3.24% to 4.92% after PEACl treatment, and further to 6.09% after GABr treatment. Table 1 summarizes the performance of all PeLEDs, demonstrating that the post-treatment can effectively enhance device electroluminescence properties. EQE histogram for devices based on GABr treatment is given in Fig. S7, indicating that the synergistic strategy we employed has good reproducibility. The electroluminescence (EL) spectra of all PeLEDs are shown in Fig. S8. Figure 5e shows that the EL peak of the PeLEDs based on PEACl and GABr treatment is located at 489 nm, with a full width at half maximum (FWHM) of approximately 20 nm, which meets the narrow spectral requirements for the display applications. Figure S9 shows their CIE coordinates, with coordinates (0.0692, 0.2563) indicating the sky blue emission. In addition, the EL peak position of the device remained unchanged when the voltage increased to 7.7 V, as shown in Fig. 5f, indicating that the blue PeLEDs exhibit excellent color stability at different voltages.

**Table 1:** Photoelectric properties of PeLEDs before and after PEACl and GABr treatments

Sample	EL peak (nm)	maximum luminance (cd/m^2^)	EQE_max_ (%)	FWHM (nm)
Control	488	875	3.24	19
PEACl	488	1876	4.92	20
PEACl + GABr	489	1325	6.09	20

To evaluate the stability of the post-processing PeLEDs, the T_50_ (defined as the time required from initial brightness to half brightness) was tested under the initial condition of 100 cd/m^2^ as shown in Fig. S10. After PEACl treatment, the T_50_ of the devices increased to approximately 9 min, mainly due to the intrinsic high stability of quasi-2D perovskites. GABr treatment further increased the T_50_ to about 11 min, resulting from the reduced defect density and improved film quality. These results collectively indicate that PEACl and GABr not only improves the electroluminescence performance of the devices but also enhances the stability.

## Conclusion

In summary, we introduced stable 2D perovskite into the 3D layer through PEACl-treatment, fabricating 2D/3D perovskite heterojunctions. To further improve the quality of perovskite film, we post deposited a layer of GABr. During the process, we systematically investigated their effects on the defect density and carrier lifetime of the films through TRPL and SCLC tests, and elucidated specific operational mechanisms of their actions. PEACl promoted Pb-X bonding formation, while GABr interacted with surface-uncoordinated Pb^2+^ via GA^+^, collectively reducing film defect density, suppressing non-radiative recombination, and enhancing radiative recombination rates. Benefiting from reduced defect density and enhanced hole injection, the performance of the device was significantly improved, achieving a maximum EQE of 6.09% and maximum luminance of 1325 cd/m^2^. Meanwhile, the moisture stability and operational stability of the devices were also significantly improved.

## Experimental methods

Materials, molecular synthesis, device fabrication, experimental procedures, and characterizations are available from Supporting Information.

## Supplementary Information

Below is the link to the electronic supplementary material.

Supplementary file1 (PDF 516 KB)

## Data Availability

The data that support the findings of this study are available from the corresponding author, upon reasonable request.

## References

[1] Chen, F., Dai, X.L., Yao, K.X., Li, Y.F., Zhang, D.S., Zhong, J.S., Liu, J., Ye, Z., He, H.: Homogeneous mono-layer mixed-halide perovskite quantum dots towards blue light-emitting diodes with stable spectra under continuous driving. Chem. Eng. J. **486**, 150435 (2024)

[2] Zhou, W., Shen, Y., Cao, L.X., Lu, Y., Tang, Y.Y., Zhang, K., Ren, H., Xie, F.M., Li, Y.Q., Tang, J.X.: Manipulating ionic behavior with bifunctional additives for efficient sky-blue perovskite light-emitting diodes. Adv. Funct. Mater. **33**(27), 2301425 (2023)

[3] Zhao, C., Wu, W., Zhan, H., Yuan, W., Li, H., Zhang, D., Wang, D., Cheng, Y., Shao, S., Qin, C., Wang, L.: Phosphonate/phosphine oxide dyad additive for efficient perovskite light-emitting diodes. Angew. Chem. **134**(13), e202117374 (2022) 10.1002/anie.202117374 35080099

[4] Li, H., Zhou, J., Tan, L., Li, M., Jiang, C., Wang, S., Zhao, X., Liu, Y., Zhang, Y., Ye, Y., Tress, W., Yi, C.: Sequential vacuum-evaporated perovskite solar cells with more than 24% efficiency. Sci. Adv. **8**(28), eabo7422 (2022) 35857518 10.1126/sciadv.abo7422 PMC10942770

[5] Jiang, J., Chu, Z., Yin, Z., Li, J., Yang, Y., Chen, J., Wu, J., You, J., Zhang, X.: Red perovskite light-emitting diodes with efficiency exceeding 25% realized by co-spacer cations. Adv. Mater. **34**(36), 2204460 (2022) 10.1002/adma.202204460 35855612

[6] Feng, W., Zhao, Y., Lin, K., Lu, J., Liang, Y., Liu, K., Xie, L., Tian, C., Lyu, T., Wei, Z.: Polymer-assisted crystal growth regulation and defect passivation for efficient perovskite light-emitting diodes. Adv. Funct. Mater. **32**(34), 2203371 (2022)

[7] Cai, W.Q., Ali, M.U., Liu, P., He, M., Zhao, C., Chen, Z.M., Zang, Y., Tang, M.C., Meng, H., Fu, H., Wei, G., Yip, H.L.: Unravelling alkali-metal-assisted domain distribution of quasi-2D perovskites for cascade energy transfer toward efficient blue light-emitting diodes. Adv. Sci. **9**(20), 2200393 (2022) 10.1002/advs.202200393 PMC9284168 35561063

[8] Zhu, Z., Wu, Y., Shen, Y., Tan, J., Shen, D., Lo, M.F., Li, M., Yuan, Y., Tang, J.X., Zhang, W., Tsang, S.W., Guan, Z., Lee, C.S.: Highly efficient sky-blue perovskite light-emitting diode via suppressing nonradiative energy loss. Chem. Mater. **33**(11), 4154–4162 (2021)

[9] Zhang, K., Zhu, N.N., Zhang, M.M., Wang, L., Xing, J.: Opportunities and challenges in perovskite LED commercialization. J. Mater. Chem. C Mater. Opt. Electron. Dev. **9**(11), 3795–3799 (2021)

[10] Zhao, L., Roh, K., Kacmoli, S., Al Kurdi, K., Jhulki, S., Barlow, S., Marder, S.R., Gmachl, C., Rand, B.P.: Thermal management enables bright and stable perovskite light-emitting diodes. Adv. Mater. **32**(25), 2000752 (2020) 10.1002/adma.202000752 32406172

[11] Zhou, C., Meng, W., Kong, L., Zhang, C., Zhang, J., Liu, F., Li, H., Jia, G., Yang, X.: Vacuum processed metal halide perovskite light-emitting diodes. Adv. Funct. Mater. **34**(8), 2307682 (2024)

[12] Du, P., Li, J., Wang, L., Sun, L., Wang, X., Xu, X., Yang, L., Pang, J., Liang, W., Luo, J., Ma, Y., Tang, J.: Efficient and large-area all vacuum-deposited perovskite light-emitting diodes via spatial confinement. Nat. Commun. **12**(1), 4751 (2021) 34362915 10.1038/s41467-021-25093-6 PMC8346511

[13] Li, J., Du, P., Guo, Q., Sun, L., Shen, Z., Zhu, J., Dong, C., Wang, L., Zhang, X., Li, L., Yang, C., Pan, J., Liu, Z., Xia, B., Xiao, Z., Du, J., Song, B., Luo, J., Tang, J.: Efficient all-thermally evaporated perovskite light-emitting diodes for active-matrix displays. Nat. Photonics **17**(5), 435–441 (2023)

[14] Ko, P.K., Ge, J., Ding, P., Chen, D., Tsang, H.L.T., Kumar, N., Halpert, J.E.: The deepest blue: major advances and challenges in deep blue emitting quasi-2D and nanocrystalline perovskite leds. Adv. Mater. **1**, 2407764 (2024) 10.1002/adma.202407764 PMC12160689 39324282

[15] Woo, S.J., Kim, J.S., Lee, T.W.: Characterization of stability and challenges to improve lifetime in perovskite LEDs. Nat. Photonics **15**(9), 630–634 (2021)

[16] Shen, Y., Shen, K.C., Li, Y.Q., Guo, M., Wang, J., Ye, Y., Xie, F.M., Ren, H., Gao, X., Song, F., Tang, J.X.: Interfacial potassium-guided grain growth for efficient deep-blue perovskite light-emitting diodes. Adv. Funct. Mater. **31**(6), 2006736 (2021)

[17] Kim, Y.C., An, H.J., Kim, D.H., Myoung, J.M., Heo, Y.J., Cho, J.H.: High-performance perovskite-based blue light-emitting diodes with operational stability by using organic ammonium cations as passivating agents. Adv. Funct. Mater. **31**(5), 2005553 (2021)

[18] Qi, H., Tong, Y., Zhang, X., Wang, H., Zhang, L., Chen, Y., Wang, Y., Shang, J., Wang, K., Wang, H.: Homogenizing energy landscape for efficient and spectrally stable blue perovskite light-emitting diodes. Adv. Mater. **36**(46), 2409319 (2024) 10.1002/adma.202409319 39302002

[19] Chen, D., Zou, G., Wu, Y., Tang, B., Rogach, A.L., Yip, H.L.: Metal halide perovskite LEDs for visible light communication and lasing applications. Adv. Mater. **1**, 2414745 (2024) 10.1002/adma.202414745 39676405

[20] Kodalle, T., Byranvand, M.M., Goudreau, M., Das, C., Roy, R., Kot, M., Briesenick, S., Zohdi, M., Rai, M., Tamura, N., Flege, J.I., Hempel, W., Sutter-Fella, C.M., Saliba, M.: An integrated deposition and passivation strategy for controlled crystallization of 2D/3D Halide perovskite films. Adv. Mater. **36**(24), 2309154 (2024) 10.1002/adma.202309154 38415385

[21] Wang, K.H., Wang, L., Liu, Y.Y., Song, Y.H., Yin, Y.C., Yao, J.S., Yang, J.N., Wang, J.J., Feng, L.Z., Zhang, Q., Zhang, Q., Yao, H.B.: High quality CsPbI_3_−Br thin films enabled by synergetic regulation of fluorine polymers and amino acid molecules for efficient pure red light emitting diodes. Adv. Opt. Mater. **9**(3), 2001684 (2021)

[22] Sun, G., Liu, X., Liu, Z., Liu, D., Meng, F., Li, Z., Chu, L., Qiu, W., Peng, X., Xie, W., Shen, C., Chen, J., Yip, H.L., Su, S.J.: Emission wavelength tuning via competing lattice expansion and octahedral tilting for efficient red perovskite light-emitting diodes. Adv. Funct. Mater. **31**(50), 2106691 (2021)

[23] Hong, Y., Yu, C., Je, H., Park, J.Y., Kim, T., Baik, H., Tomboc, G.M., Kim, Y., Ha, J.M., Joo, J., Kim, C.W., Woo, H.Y., Park, S., Choi, D.H., Lee, K.: Perovskite nanocrystals protected by hermetically sealing for highly bright and stable deep-blue light-emitting diodes. Adv. Sci. **10**(23), 2302906 (2023) 10.1002/advs.202302906 PMC10427390 37271888

[24] Pang, H., Du, S., Deng, J., Kong, W., Zhao, Y., Zheng, B., Ma, L.: Enhancing carrier transport in 2D/3D perovskite heterostructures through organic cation fluorination. Small **20**(34), 2401797 (2024) 10.1002/smll.202401797 38577831

[25] Fakharuddin, A., Qiu, W., Croes, G., Devižis, A., Gegevičius, R., Vakhnin, A., Rolin, C., Genoe, J., Gehlhaar, R., Kadashchuk, A., Gulbinas, V., Heremans, P.: Reduced efficiency roll-off and improved stability of mixed 2D/3D perovskite light emitting diodes by balancing charge injection. Adv. Funct. Mater. **29**(37), 1904101 (2019)

[26] Liu, Z., Qiu, W., Peng, X., Sun, G., Liu, X., Liu, D., Li, Z., He, F., Shen, C., Gu, Q., Ma, F., Yip, H.L., Hou, L., Qi, Z., Su, S.J.: Perovskite light-emitting diodes with EQE exceeding 28% through a synergetic dual-additive strategy for defect passivation and nanostructure regulation. Adv. Mater. **33**(43), 2103268 (2021) 10.1002/adma.202103268 34545631

[27] Bai, F., Zhang, J., Yuan, Y., Liu, H., Li, X., Chueh, C.C., Yan, H., Zhu, Z., Jen, A.K.Y.: A 0D/3D heterostructured all-inorganic halide perovskite solar cell with high performance and enhanced phase stability. Adv. Mater. **31**(48), 1904735 (2019) 10.1002/adma.201904735 31608506

[28] Li, Z., Liu, X., Xu, J., Yang, S., Zhao, H., Huang, H., Liu, S.F., Yao, J.: All-inorganic 0D/3D Cs_4_Pb(IBr)_6_/CsPbI_3−__*x*_Br_*x*_ mixed-dimensional perovskite solar cells with enhanced efficiency and stability. J. Mater. Chem. C Mater. Opt. Electron. Dev. **8**(21), 6977–6987 (2020)

[29] Mahmud, M.A., Pham, H.T., Duong, T., Yin, Y., Peng, J., Wu, Y., Liang, W., Li, L., Kumar, A., Shen, H., Walter, D., Nguyen, H.T., Mozaffari, N., Tabi, G.D., Andersson, G., Catchpole, K.R., Weber, K.J., White, T.P.: Combined bulk and surface passivation in dimensionally engineered 2D–3D perovskite films via chlorine diffusion. Adv. Funct. Mater. **31**(46), 2104251 (2021)

[30] Cui, Z., Li, W., Feng, B., Li, Y., Guo, X., Yuan, H., Weng, Q., You, T., Zhang, W., Li, X., Fang, J.: Substrate induced p–n transition for inverted perovskite solar cells. Adv. Mater. **36**(41), 2410273 (2024) 10.1002/adma.202410273 39148185

[31] Zhou, X., Zhang, L., Wang, X., Liu, C., Chen, S., Zhang, M., Li, X., Yi, W., Xu, B.: Highly efficient and stable gabr-modified ideal-bandgap (1.35 ev) sn/pb perovskite solar cells achieve 20.63% efficiency with a record small Voc deficit of 0.33 V. Adv. Mater. **32**(14), 1908107 (2020) 10.1002/adma.201908107 32100401

[32] Zhang, F., Cai, B., Song, J., Han, B., Zhang, B., Zeng, H.: Efficient blue perovskite light-emitting diodes boosted by 2D/3D energy cascade channels. Adv. Funct. Mater. **30**(27), 2001732 (2020)

[33] Han, T.H., Tan, S., Xue, J., Meng, L., Lee, J.W., Yang, Y.: Interface and defect engineering for metal halide perovskite optoelectronic devices. Adv. Mater. **31**(47), 1803515 (2019) 10.1002/adma.201803515 30761623

[34] Tzoganakis, N., Spiliarotis, E., Tsikritzis, D., Kymakis, E.: 4F-phenethylammonium chloride as a key component for interfacial engineering of wide-bandgap perovskite absorber. Nano Energy **128**, 109914 (2024)

[35] Cui, X., Wang, P., Shi, B., Zhao, Y., Zhang, X.: Insights into the effect of bromine-based organic salts on the efficiency and stability of wide bandgap perovskite. Nano Select **2**(3), 615–623 (2021)

[36] Gong, C., Wang, X., Xia, X., Yang, X., Wang, L., Li, F.: In-situ guanidinium bromide passivation treatment of CsPbBr 3 perovskite quantum dots exhibiting high photoluminescence and environmental stability. Appl. Surf. Sci. **559**, 149986 (2021)

[37] Tang, L., Wang, X., Liu, X., Zhang, J., Wang, S., Zhao, Y., Gong, J., Li, J., Xiao, X.: Mixed solvents assisted post-treatment enables high-efficiency single-junction perovskite and 4T Perovskite/CIGS tandem solar cells. Adv. Sci. **9**, 2201768 (2022) 10.1002/advs.202201768 PMC9376828 35673955

[38] Kim, J.S., Heo, J.M., Park, G.S., Woo, S.J., Cho, C., Yun, H.J., Kim, D.H., Park, J., Lee, S.C., Park, S.H., Yoon, E., Greenham, N.C., Lee, T.W.: Ultra-bright, efficient and stable perovskite light-emitting diodes. Nature **611**(7937), 688–694 (2022) 36352223 10.1038/s41586-022-05304-w

[39] Dong, Y., Zhu, R., Jia, Y.: Linear relationship between the dielectric constant and band gap in low-dimensional mixed-halide perovskites. J. Phys. Chem. C **125**(27), 14883–14890 (2021)

[40] Huang, J., Tan, S., Lund, P.D., Zhou, H.: Impact of H_2_O on organic–inorganic hybrid perovskite solar cells. Energy Environ. Sci. **10**(11), 2284–2311 (2017)

[41] Azmi, R., Ugur, E., Seitkhan, A., Aljamaan, F., Subbiah, A.S., Liu, J., Harrison, G.T., Nugraha, M.I., Eswaran, M.K., Babics, M., Chen, Y., Xu, F., Allen, T.G., Rehman, A., Wang, C.L., Anthopoulos, T.D., Schwingenschlögl, U., De Bastiani, M., Aydin, E., De Wolf, S.: Damp heat–stable perovskite solar cells with tailored-dimensionality 2D/3D heterojunctions. Science **376**(6588), 73–77 (2022) 35175829 10.1126/science.abm5784

