# Supplementary material for: Improved vacuum-evaporated blue perovskite light-emitting diodes with phenethylammonium chloride and guanidinium bromide synergistic post-processing modification
Source: Front Optoelectron. 2025 Mar 24;18(1):5. doi: 10.1007/s12200-025-00152-8 (PMC11930901; doi:10.1007/s12200-025-00152-8)
Supplement: Supplementary file 1 — Supplementary file1 (PDF 516 KB) [file 12200_2025_152_MOESM1_ESM.pdf]

Supporting Information

Improved vacuum-evaporated blue perovskite light-emitting diodes with phenethylammonium chloride and guanidinium bromide synergistic post-processing modification

Liang Sun, Xiping He, Zhiyuan He, Feihu Zhang, Chencheng Peng, Ben Chen, Runda Guo*, Lei Wang*

Wuhan National Laboratory for Optoelectronics, School of Optical and Electronic Information, Huazhong University of Science and Technology, Wuhan 430074, China

Email: wanglei@mail.hust.edu.cn (Lei Wang); runda_guo@hust.edu.cn (Runda Guo)

Experimental Section

Materials: CsBr (99.999%, Sigma – Aldrich), CsCl (99.999%, Sigma – Aldrich), $PbBr_2$ (99.999%, Sigma–Aldrich), PVK (Mn≈25000-50000, 99.9%, Sigma–Aldrich), PEACl (99.5%, Xi'an Polymer Light Technology Corporation), GABr (99.8%, J&K Scientific), $Ni(CH_3COO)_2{\cdot}4H_2O$ (99.9%, Across organics), TPBi (99.9%, Xi'an Polymer Light Technology Corporation), LiF (99.9%, Aladdin-reagent), Acetone (99.7%, Sinopharm Chemical Reagent Co.,Ltd), isopropanol (99.5%, Sigma–Aldrich). All of the reagents were directly used as received without any further purifications.

Preparation of 2D/3D Perovskite Films: To maintain the environmental atmosphere of the vacuum deposition chamber and reduce impurities in the chamber atmosphere, the deposition chamber should be baked at a high temperature before depositing perovskite precursor materials. Additionally, to ensure the stability of deposition rates, the ratio factor of each precursor source must be calibrated before deposition, ensuring stable deposition rates throughout the process to achieve uniform perovskite films and enhance film quality. During deposition, the substrate rotation speed should be kept at 8 r/min to evenly distribute the precursor on the substrate. The substrate can be opened for deposition when the chamber vacuum is lower than 10^{-4} Pa. The evaporative rate ratio

of precursor materials $PbBr_2$, CsBr, and CsCl is 1:0.85:0.55, and the temperature of the sample base should be maintained at -10°C.

PeLEDs Fabrication: The ITO glass substrate (procured from Liaoning Advanced Election T echnology Co) used for device fabrication needs to be cleaned by placing it on a dedicated ITO cleaning rack. It underwent ultrasonic cleaning with specialized ITO cleaning solution, deionized water, acetone and isopropanol, each for 1 h. After cleaning, it was dried with a high-pressure nitrogen gun and then dried in an oven at 120 ℃. Following drying, the substrate was placed face-up in a UV ozone cleaner for 30 min. NiO_x was spin-coated on the ITO substrate using the static spin-coating method at 4000 rpm for 30 s, followed by annealing for 45 min in air at 350 °C. After cooling, the NiO_x-coated substrate was transferred to a glove box filled with N_2, PVK-chlorobenzene solution (4 mg/mL) was sprayed onto the NiO_x-coated substrate at 4000 rpm for 30 s and annealed on a hotplate at 170 °C for 30 min. Subsequently, the substrate was transferred to the deposition chamber to deposit a 100 nm thick perovskite film. PEACl post-processing was conducted in the glove box before returning to the deposition chamber to deposit 5 nm GABr at a rate of 0.1 Å/s. The substrate was transferred to the chamber for vaporization of the transport layer material using a mechanical transfer bar to deposit 20 nm TPBi (at a rate of 1 Å/s), 1 nm LiF (at a rate of 0.1 Å/s), and 100 nm Al in sequence. After annealing at 60°C for 20 min, the encapsulated sheet was sealed onto the effective area of the device by using a UV curing adhesive, resulting in the fabrication of PeLEDs.

Characterization of Perovskite Films and Devices: The ultraviolet-visible absorption spectra were measured by UV visible spectrophotometer (Shimadzu UV-3600). The steady-state PL spectra were obtained under the irradiation of a 350 nm Xe lamp using a PR655 optical research spectroscopy scanner. TRPL spectra were tested by an Edinburgh FLS920 time-dependent single photon counter. XRD spectra were measured using an Empyrean X-ray diffractometer with a Cu Kα source. SEM images were obtained using a scanning electron microscope (GeminiSEM 300). AFM images were obtained in tapping mode using a Bruker RTESPA-300 probe with an atomic force microscope (SPM9700). SCLC data were gained by measuring the current density of single-carrier devices at different voltages using a Keithley 2400. UPS spectrum were measured using an X-ray photoelectron spectrometer (AXIS-ULTRA DLD-600W). XPS spectra were tested using monochromatic Mg Kα radiation as an excitation source. The current density-voltage-luminance

characteristics and EL spectrum of the device were measured using a Keithley 2400 source meter equipped with a PR655 spectroradiometer.

Note S1

The carrier lifetimes associated with defects were extracted by fitting the decay curves, as shown in the following fitting **Equation S1**:[1]

$$Y = A_1\exp(-t/\tau_1) + A_2\exp(-t/\tau_2) + y_0 \qquad (1)$$

Where τ_1 and τ_2 represent the defect recombination and radiative recombination, respectively, and A_1 and A_2 represent the proportions of each lifetime in the total lifetime. The average lifetime τ_{ave} can be determined from the **Equation S2**:

$$\tau_{ave} = (A_1\tau_1^2 + A_2\tau_2^2) / (A_1\tau_1 + A_2\tau_2) \qquad (2)$$

Note S2

The work functions of the original film, PEACl-treated, and GABr-treated films can be calculated from the **Equation S3**:

$$W = h\upsilon - E_{cut\text{-}off} \qquad (3)$$

Where W represents the work function, $h\upsilon$ denotes the photon energy, which is 21.2 eV.

The values of the valence band maximum of the original film, PEACl-treated, and GABr-treated films can be obtained from the **Equation S4**:

$$E_{VB} = -W - E_2 \qquad (4)$$

Where E_{VB} is the value of the valence band maximum.

Note S3

As shown in **Figure S3**, the cut-off energies ($E_{cut\text{-}off}$) obtained from the high binding energy secondary electron cutoff edge in the UPS spectra are 16.42 eV, 16.63 eV, and 16.21 eV for the original film, PEACl-treated, and GABr-treated films, respectively. Subsequently, according to the **Equation S3**[2], the work functions of the original film, PEACl-treated, and GABr-treated films can be calculated as 4.78 eV, 4.57 eV, and 4.99 eV, respectively. Furthermore, by analyzing the valence band region in UPS spectra, the E_2 values for the original film, PEACl-treated, and GABr-treated films are determined to be 1.02 eV, 1.07 eV, and 1.04 eV. The values of the valence band

maximum of the original film, PEACl-treated, and GABr-treated films can be obtained as 5.80 eV, 5.67 eV, and 6.03 eV, respectively, based on the Equation S4 and the value of E_2.

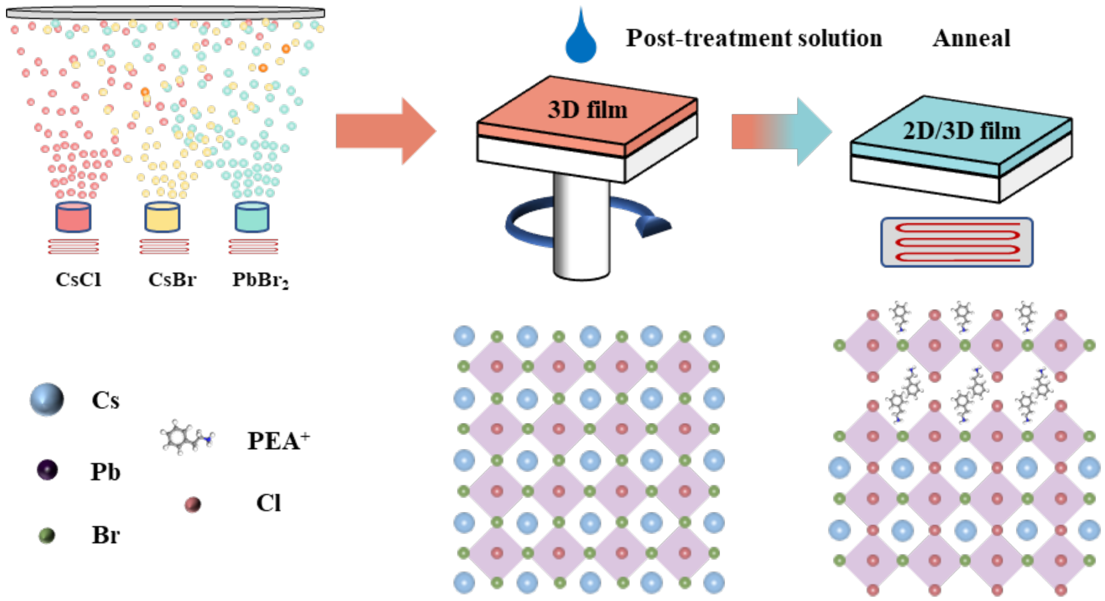

Figure S1. Schematic of 2D/3D heterojunction preparation process and structure.

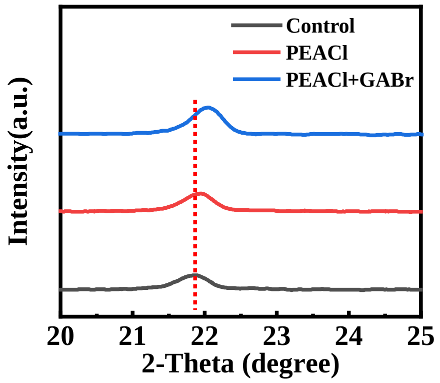

Figure S2. The magnification of the XRD patterns for perovskite films.

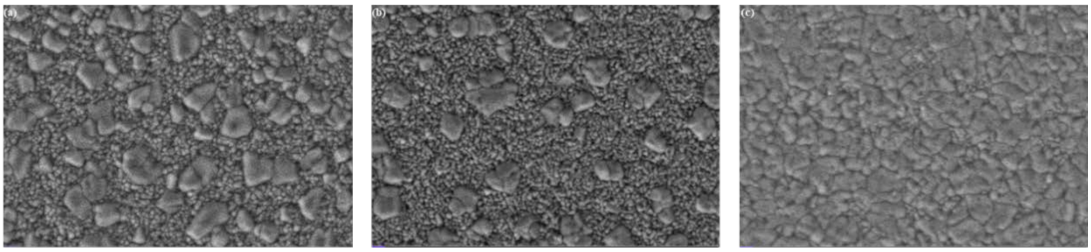

Figure S3. The SEM image of perovskite films for a) control, b) PEACl-modified and c) PEACl and GABr treatments.

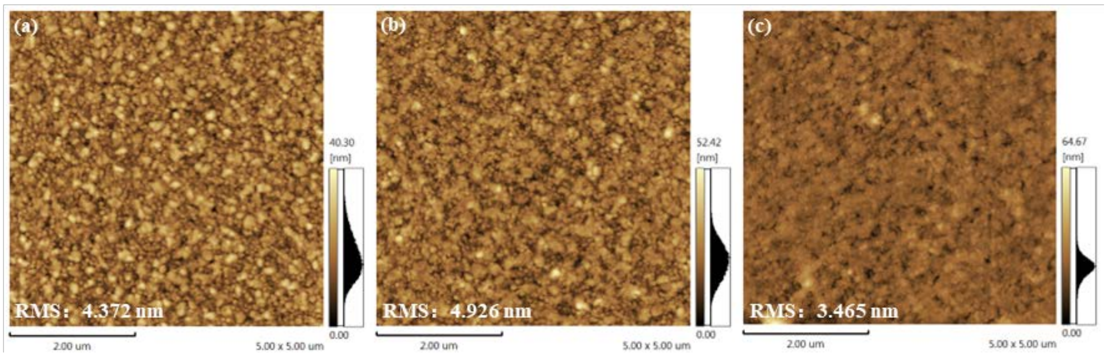

Figure S4. The AFM of perovskite films for a) control, b) PEACl-modified and c) PEACl and GABr treatment.

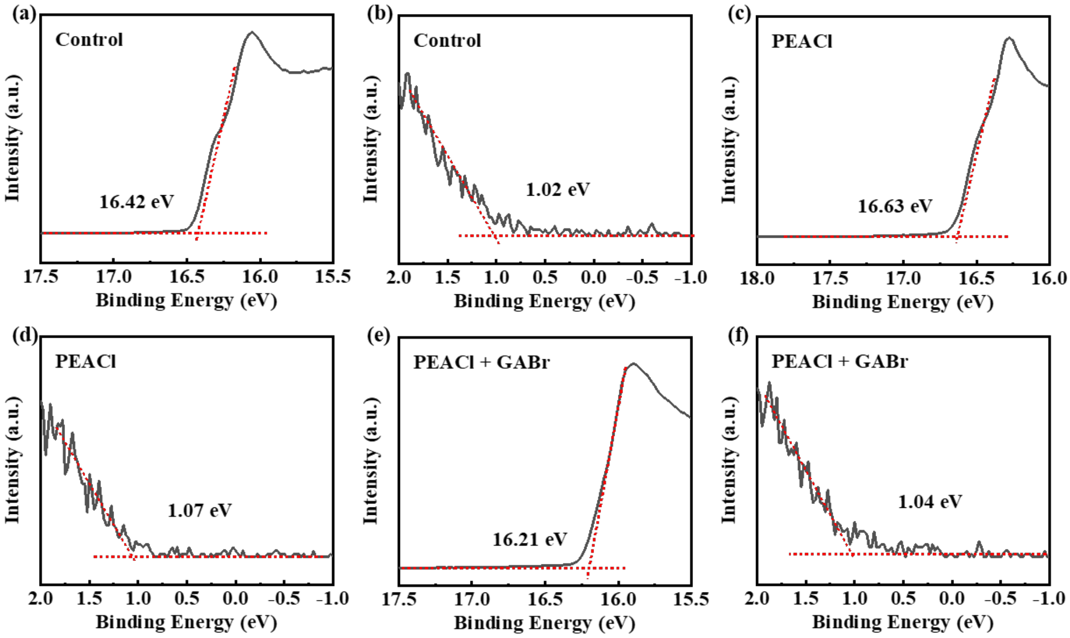

Figure S5. The UPS spectrum of the pristine film for a) Secondary electron cut-off region. b) Valence band cut-off region. The UPS spectrum of the PEACl-treated film for c) Secondary electron cut-off region. d) Valence band cut-off region. The UPS spectrum of the film after PEACl and GABr treatments for e) Secondary electron cut-off region. f) Valence band cut-off region.

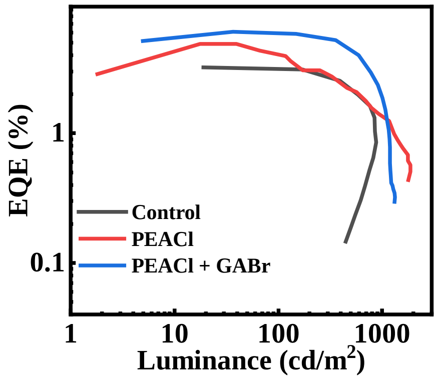

Figure S6. EQE–luminance curves for PeLEDs.

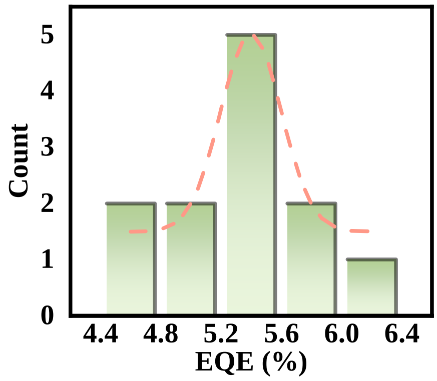

Figure S7. EQE histograms for devices based on GABr treatment.

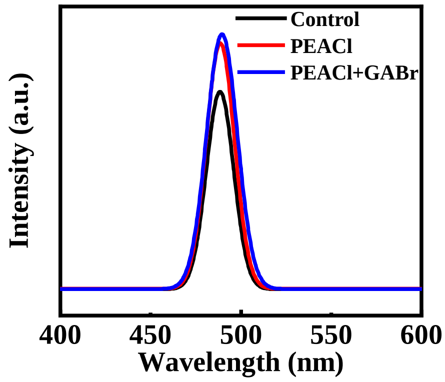

Figure S8. The EL spectra of all PeLEDs.

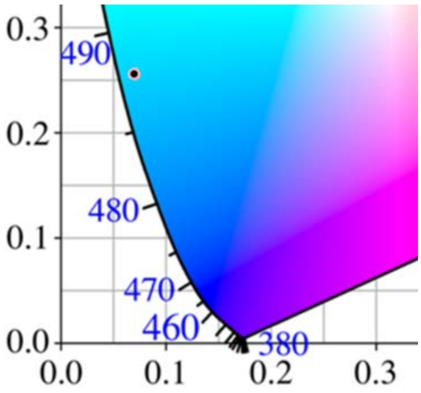

Figure S9. The CIE coordinates of post-processed PeLEDs.

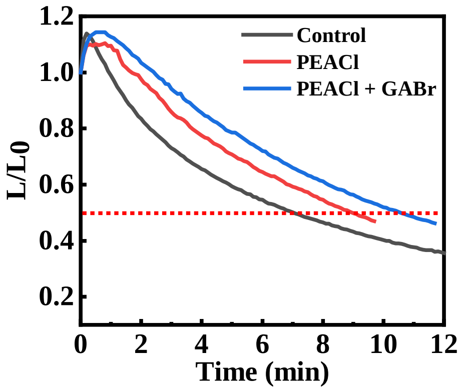

Figure S10. Operational stability of PeLEDs before and after PEACl and GABr treatments.

Table S1. The TRPL fitting results of films before and after treatment with PEACl and GABr.

Sample	A_1	τ_1 (ns)	A_2	τ_2 (ns)	τ_{ave} (ns)
Control	4.29	0.52	0.18	2.42	0.83
PEACl	1.63	1.27	0.31	5.90	3.44
PEACl+GABr	0.81	2.10	0.34	10.38	7.69

References

[1] Z. Liu, W. Qiu, X. Peng, G. Sun, X. Liu, D. Liu, et al. Perovskite light-emitting diodes with EQE exceeding 28% through a synergetic dual-additive strategy for defect passivation and nanostructure regulation. Advanced Materials. 2021, 33(43): 2103268

[2] F. Zhang, S. Y. Park, C. Yao, H. Lu, S. P. Dunfield, C. Xiao, et al. Metastable Dion-Jacobson 2D structure enables efficient and stable perovskite solar cells. Science. 2022, 375(6576): 71-76
